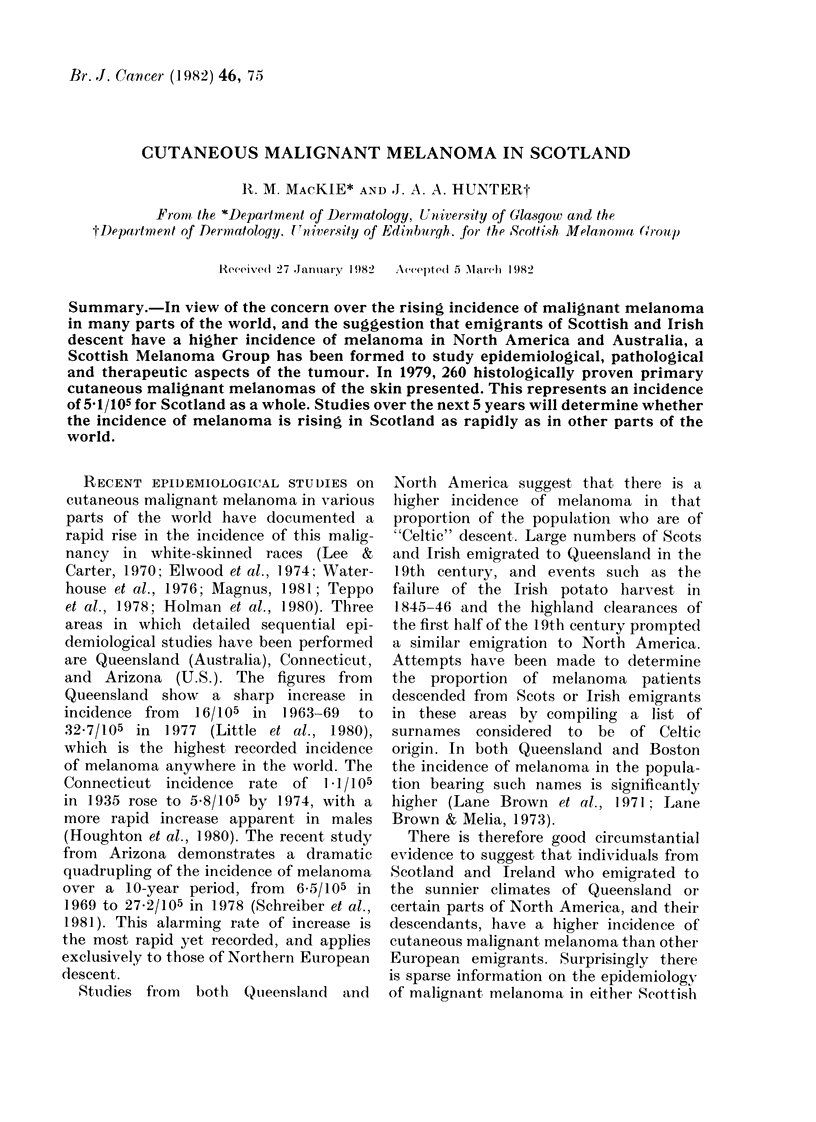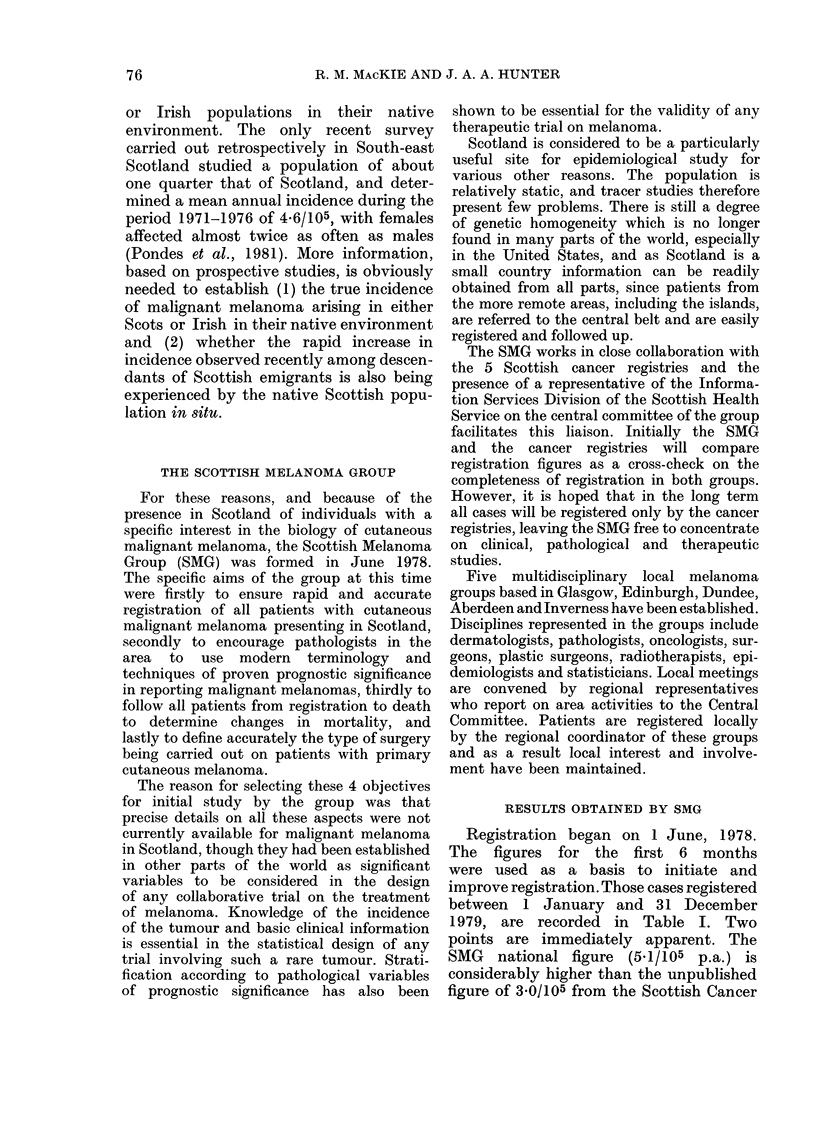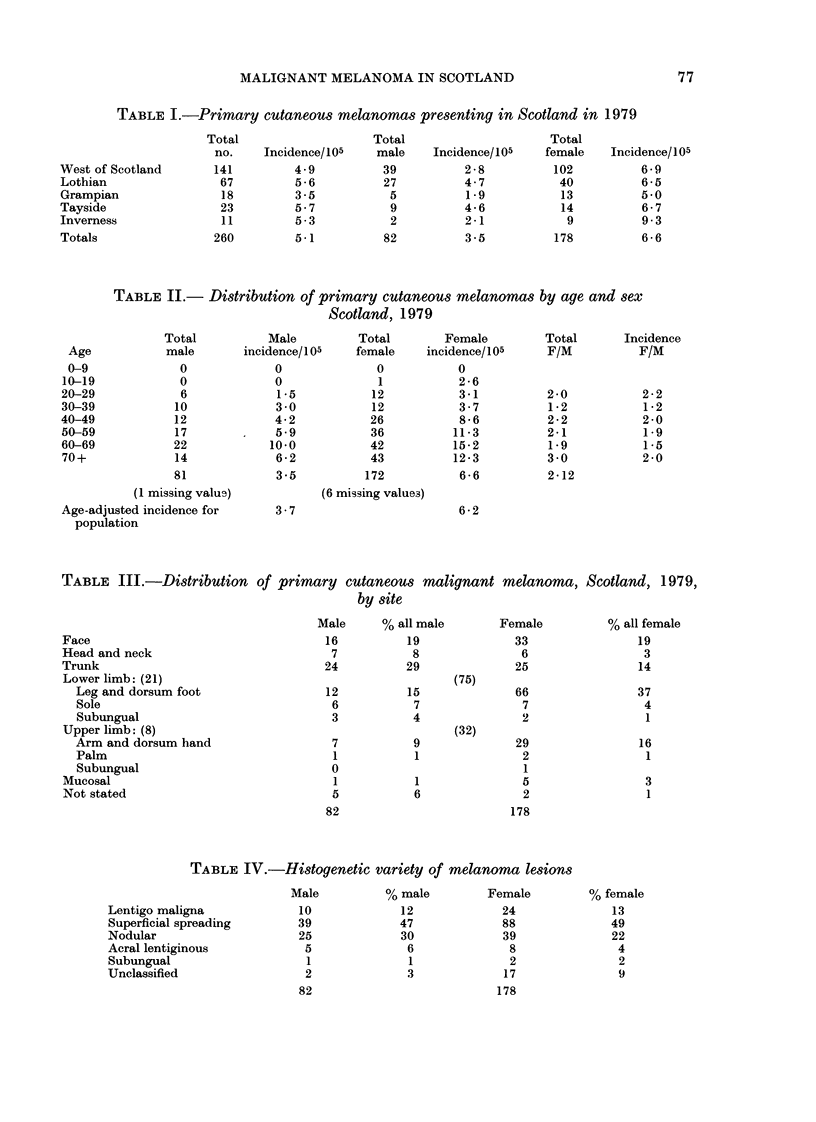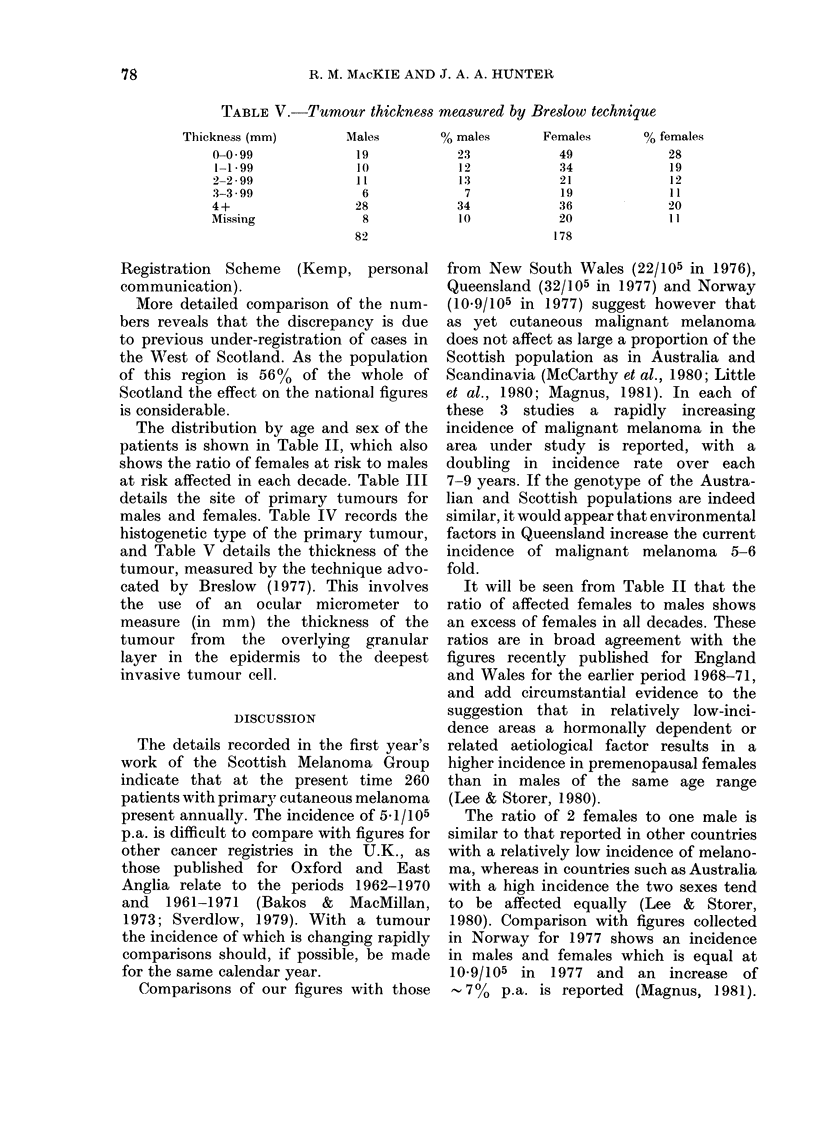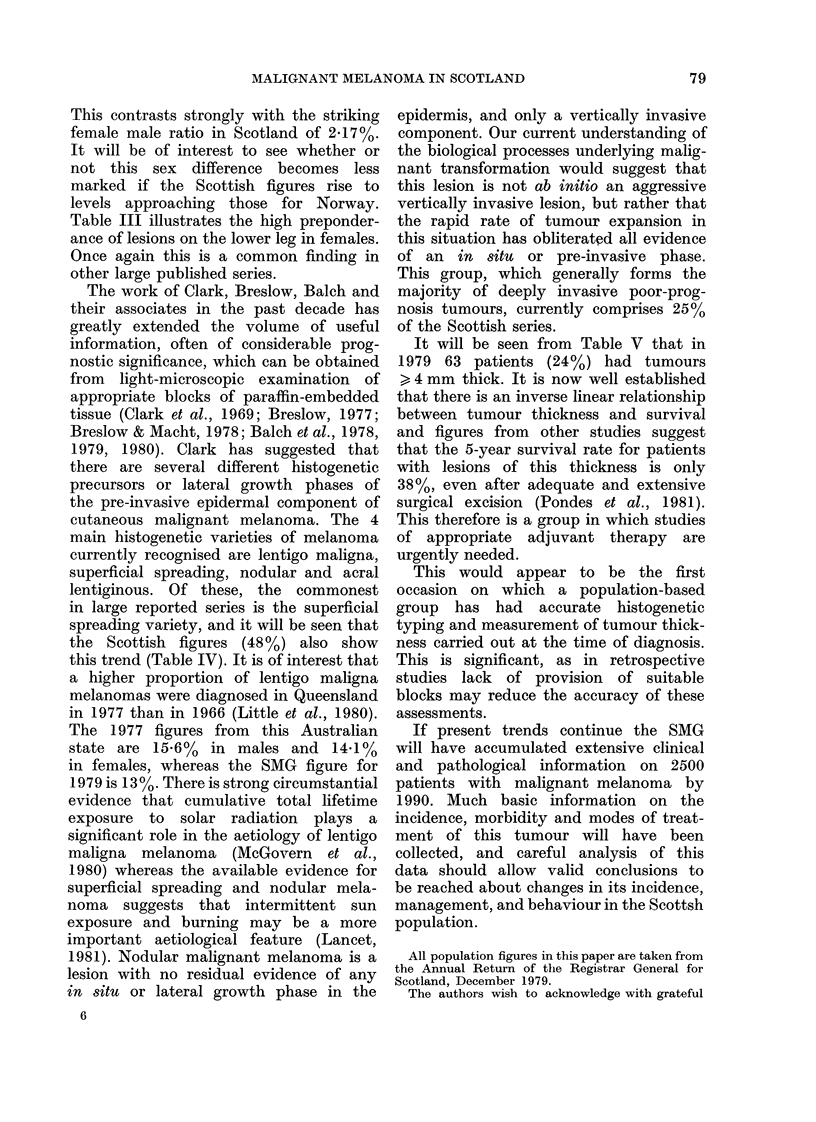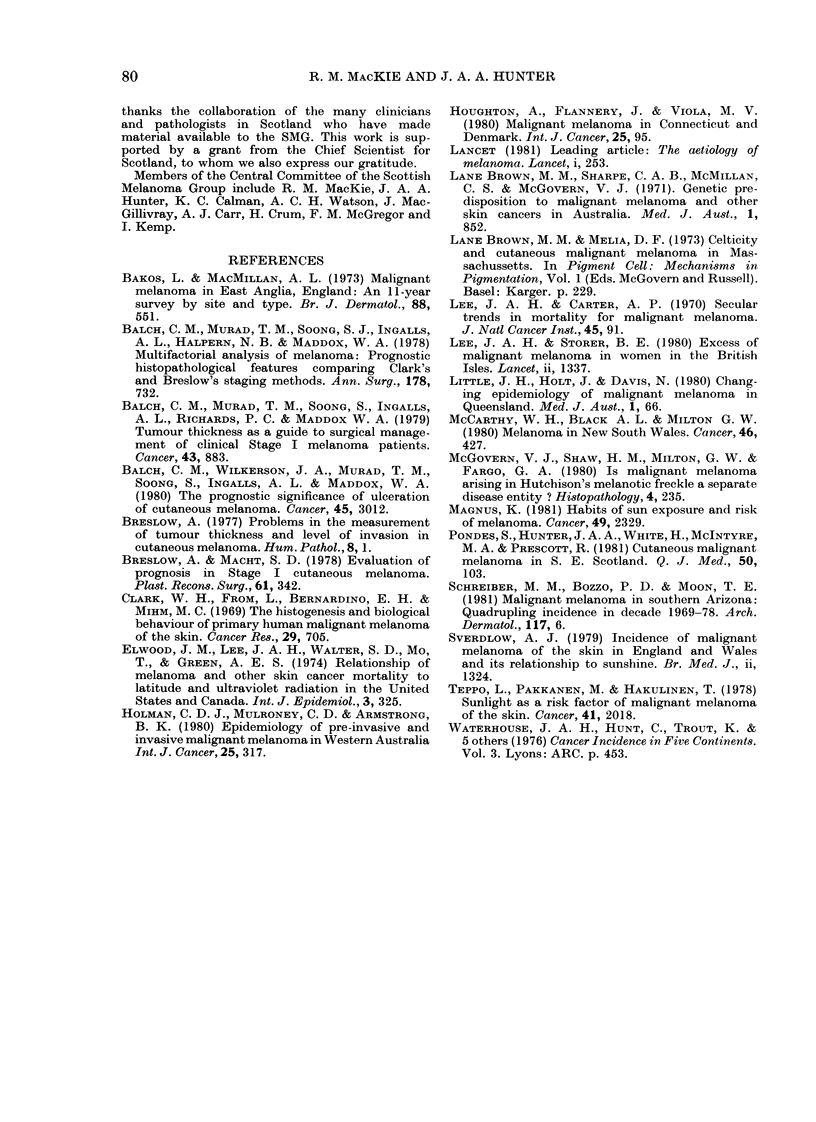# Cutaneous malignant melanoma in Scotland.

**DOI:** 10.1038/bjc.1982.167

**Published:** 1982-07

**Authors:** R. M. MacKie, J. A. Hunter

## Abstract

In view of the concern over the rising incidence of malignant melanoma in many parts of the world, and the suggestion that emigrants of Scottish and Irish descent have a higher incidence of melanoma in North America and Australia, a Scottish Melanoma Group has been formed to study epidemiological, pathological and therapeutic aspects of the tumour. In 1979, 260 histologically proven primary cutaneous malignant melanomas of the skin presented. This represents an incidence of 5.1/10(5) for Scotland as a whole. Studies over the next 5 years will determine whether the incidence of melanoma is rising in Scotland as rapidly as in other parts of the world.


*Br. J. Cancer* (1982) **46**, 75

### CUTANEOUS MALIGNANT MELANOMA IN SCOTLAND

R. M. MACKIE* AND J. A. A. HUNTER†

*From the *Department of Dermatology, University of Glasgow and the †Department of Dermatology, University of Edinburgh, for the Scottish Melanoma Group*

Received 27 January 1982 Accepted 5 March 1982

**Summary.—In view of the concern over the rising incidence of malignant melanoma in many parts of the world, and the suggestion that emigrants of Scottish and Irish descent have a higher incidence of melanoma in North America and Australia, a Scottish Melanoma Group has been formed to study epidemiological, pathological and therapeutic aspects of the tumour. In 1979, 260 histologically proven primary cutaneous malignant melanomas of the skin presented. This represents an incidence of $5{\cdot}1/10^5$ for Scotland as a whole. Studies over the next 5 years will determine whether the incidence of melanoma is rising in Scotland as rapidly as in other parts of the world.**

RECENT EPIDEMIOLOGICAL STUDIES on cutaneous malignant melanoma in various parts of the world have documented a rapid rise in the incidence of this malignancy in white-skinned races (Lee & Carter, 1970; Elwood *et al.*, 1974; Waterhouse *et al.*, 1976; Magnus, 1981; Teppo *et al.*, 1978; Holman *et al.*, 1980). Three areas in which detailed sequential epidemiological studies have been performed are Queensland (Australia), Connecticut, and Arizona (U.S.). The figures from Queensland show a sharp increase in incidence from $16/10^5$ in 1963–69 to $32{\cdot}7/10^5$ in 1977 (Little *et al.*, 1980), which is the highest recorded incidence of melanoma anywhere in the world. The Connecticut incidence rate of $1{\cdot}1/10^5$ in 1935 rose to $5{\cdot}8/10^5$ by 1974, with a more rapid increase apparent in males (Houghton *et al.*, 1980). The recent study from Arizona demonstrates a dramatic quadrupling of the incidence of melanoma over a 10-year period, from $6{\cdot}5/10^5$ in 1969 to $27{\cdot}2/10^5$ in 1978 (Schreiber *et al.*, 1981). This alarming rate of increase is the most rapid yet recorded, and applies exclusively to those of Northern European descent.

Studies from both Queensland and North America suggest that there is a higher incidence of melanoma in that proportion of the population who are of "Celtic" descent. Large numbers of Scots and Irish emigrated to Queensland in the 19th century, and events such as the failure of the Irish potato harvest in 1845–46 and the highland clearances of the first half of the 19th century prompted a similar emigration to North America. Attempts have been made to determine the proportion of melanoma patients descended from Scots or Irish emigrants in these areas by compiling a list of surnames considered to be of Celtic origin. In both Queensland and Boston the incidence of melanoma in the population bearing such names is significantly higher (Lane Brown *et al.*, 1971; Lane Brown & Melia, 1973).

There is therefore good circumstantial evidence to suggest that individuals from Scotland and Ireland who emigrated to the sunnier climates of Queensland or certain parts of North America, and their descendants, have a higher incidence of cutaneous malignant melanoma than other European emigrants. Surprisingly there is sparse information on the epidemiology of malignant melanoma in either Scottish or Irish populations in their native environment. The only recent survey carried out retrospectively in South-east Scotland studied a population of about one quarter that of Scotland, and determined a mean annual incidence during the period 1971–1976 of $4{\cdot}6/10^5$, with females affected almost twice as often as males (Pondes *et al.*, 1981). More information, based on prospective studies, is obviously needed to establish (1) the true incidence of malignant melanoma arising in either Scots or Irish in their native environment and (2) whether the rapid increase in incidence observed recently among descendants of Scottish emigrants is also being experienced by the native Scottish population *in situ*.

### THE SCOTTISH MELANOMA GROUP

For these reasons, and because of the presence in Scotland of individuals with a specific interest in the biology of cutaneous malignant melanoma, the Scottish Melanoma Group (SMG) was formed in June 1978. The specific aims of the group at this time were firstly to ensure rapid and accurate registration of all patients with cutaneous malignant melanoma presenting in Scotland, secondly to encourage pathologists in the area to use modern terminology and techniques of proven prognostic significance in reporting malignant melanomas, thirdly to follow all patients from registration to death to determine changes in mortality, and lastly to define accurately the type of surgery being carried out on patients with primary cutaneous melanoma.

The reason for selecting these 4 objectives for initial study by the group was that precise details on all these aspects were not currently available for malignant melanoma in Scotland, though they had been established in other parts of the world as significant variables to be considered in the design of any collaborative trial on the treatment of melanoma. Knowledge of the incidence of the tumour and basic clinical information is essential in the statistical design of any trial involving such a rare tumour. Stratification according to pathological variables of prognostic significance has also been shown to be essential for the validity of any therapeutic trial on melanoma.

Scotland is considered to be a particularly useful site for epidemiological study for various other reasons. The population is relatively static, and tracer studies therefore present few problems. There is still a degree of genetic homogeneity which is no longer found in many parts of the world, especially in the United States, and as Scotland is a small country information can be readily obtained from all parts, since patients from the more remote areas, including the islands, are referred to the central belt and are easily registered and followed up.

The SMG works in close collaboration with the 5 Scottish cancer registries and the presence of a representative of the Information Services Division of the Scottish Health Service on the central committee of the group facilitates this liaison. Initially the SMG and the cancer registries will compare registration figures as a cross-check on the completeness of registration in both groups. However, it is hoped that in the long term all cases will be registered only by the cancer registries, leaving the SMG free to concentrate on clinical, pathological and therapeutic studies.

Five multidisciplinary local melanoma groups based in Glasgow, Edinburgh, Dundee, Aberdeen and Inverness have been established. Disciplines represented in the groups include dermatologists, pathologists, oncologists, surgeons, plastic surgeons, radiotherapists, epidemiologists and statisticians. Local meetings are convened by regional representatives who report on area activities to the Central Committee. Patients are registered locally by the regional coordinator of these groups and as a result local interest and involvement have been maintained.

### RESULTS OBTAINED BY SMG

Registration began on 1 June, 1978. The figures for the first 6 months were used as a basis to initiate and improve registration. Those cases registered between 1 January and 31 December 1979, are recorded in Table I. Two points are immediately apparent. The SMG national figure ($5{\cdot}1/10^5$ p.a.) is considerably higher than the unpublished figure of $3{\cdot}0/10^5$ from the Scottish Cancer

TABLE I.—*Primary cutaneous melanomas presenting in Scotland in* 1979

| | Total no. | Incidence/$10^5$ | Total male | Incidence/$10^5$ | Total female | Incidence/$10^5$ |
|---|---|---|---|---|---|---|
| West of Scotland | 141 | 4·9 | 39 | 2·8 | 102 | 6·9 |
| Lothian | 67 | 5·6 | 27 | 4·7 | 40 | 6·5 |
| Grampian | 18 | 3·5 | 5 | 1·9 | 13 | 5·0 |
| Tayside | 23 | 5·7 | 9 | 4·6 | 14 | 6·7 |
| Inverness | 11 | 5·3 | 2 | 2·1 | 9 | 9·3 |
| Totals | 260 | 5·1 | 82 | 3·5 | 178 | 6·6 |

TABLE II.— *Distribution of primary cutaneous melanomas by age and sex Scotland,* 1979

| Age | Total male | Male incidence/$10^5$ | Total female | Female incidence/$10^5$ | Total F/M | Incidence F/M |
|---|---|---|---|---|---|---|
| 0–9 | 0 | 0 | 0 | 0 | | |
| 10–19 | 0 | 0 | 1 | 2·6 | | |
| 20–29 | 6 | 1·5 | 12 | 3·1 | 2·0 | 2·2 |
| 30–39 | 10 | 3·0 | 12 | 3·7 | 1·2 | 1·2 |
| 40–49 | 12 | 4·2 | 26 | 8·6 | 2·2 | 2·0 |
| 50–59 | 17 | 5·9 | 36 | 11·3 | 2·1 | 1·9 |
| 60–69 | 22 | 10·0 | 42 | 15·2 | 1·9 | 1·5 |
| 70+ | 14 | 6·2 | 43 | 12·3 | 3·0 | 2·0 |
| | 81 | 3·5 | 172 | 6·6 | 2·12 | |
| | (1 missing value) | | (6 missing values) | | | |
| Age-adjusted incidence for population | | 3·7 | | 6·2 | | |

TABLE III.—*Distribution of primary cutaneous malignant melanoma, Scotland,* 1979, *by site*

| | Male | % all male | | Female | % all female |
|---|---|---|---|---|---|
| Face | 16 | 19 | | 33 | 19 |
| Head and neck | 7 | 8 | | 6 | 3 |
| Trunk | 24 | 29 | | 25 | 14 |
| Lower limb: (21) | | | (75) | | |
| Leg and dorsum foot | 12 | 15 | | 66 | 37 |
| Sole | 6 | 7 | | 7 | 4 |
| Subungual | 3 | 4 | | 2 | 1 |
| Upper limb: (8) | | | (32) | | |
| Arm and dorsum hand | 7 | 9 | | 29 | 16 |
| Palm | 1 | 1 | | 2 | 1 |
| Subungual | 0 | | | 1 | |
| Mucosal | 1 | 1 | | 5 | 3 |
| Not stated | 5 | 6 | | 2 | 1 |
| | 82 | | | 178 | |

TABLE IV.—*Histogenetic variety of melanoma lesions*

| | Male | % male | Female | % female |
|---|---|---|---|---|
| Lentigo maligna | 10 | 12 | 24 | 13 |
| Superficial spreading | 39 | 47 | 88 | 49 |
| Nodular | 25 | 30 | 39 | 22 |
| Acral lentiginous | 5 | 6 | 8 | 4 |
| Subungual | 1 | 1 | 2 | 2 |
| Unclassified | 2 | 3 | 17 | 9 |
| | 82 | | 178 | |

TABLE V.—*Tumour thickness measured by Breslow technique*

| Thickness (mm) | Males | % males | Females | % females |
|---|---|---|---|---|
| 0–0·99 | 19 | 23 | 49 | 28 |
| 1–1·99 | 10 | 12 | 34 | 19 |
| 2–2·99 | 11 | 13 | 21 | 12 |
| 3–3·99 | 6 | 7 | 19 | 11 |
| 4+ | 28 | 34 | 36 | 20 |
| Missing | 8 | 10 | 20 | 11 |
| | 82 | | 178 | |

Registration Scheme (Kemp, personal communication).

More detailed comparison of the numbers reveals that the discrepancy is due to previous under-registration of cases in the West of Scotland. As the population of this region is 56% of the whole of Scotland the effect on the national figures is considerable.

The distribution by age and sex of the patients is shown in Table II, which also shows the ratio of females at risk to males at risk affected in each decade. Table III details the site of primary tumours for males and females. Table IV records the histogenetic type of the primary tumour, and Table V details the thickness of the tumour, measured by the technique advocated by Breslow (1977). This involves the use of an ocular micrometer to measure (in mm) the thickness of the tumour from the overlying granular layer in the epidermis to the deepest invasive tumour cell.

### DISCUSSION

The details recorded in the first year's work of the Scottish Melanoma Group indicate that at the present time 260 patients with primary cutaneous melanoma present annually. The incidence of $5{\cdot}1/10^5$ p.a. is difficult to compare with figures for other cancer registries in the U.K., as those published for Oxford and East Anglia relate to the periods 1962–1970 and 1961–1971 (Bakos & MacMillan, 1973; Sverdlow, 1979). With a tumour the incidence of which is changing rapidly comparisons should, if possible, be made for the same calendar year.

Comparisons of our figures with those from New South Wales ($22/10^5$ in 1976), Queensland ($32/10^5$ in 1977) and Norway ($10{\cdot}9/10^5$ in 1977) suggest however that as yet cutaneous malignant melanoma does not affect as large a proportion of the Scottish population as in Australia and Scandinavia (McCarthy *et al.*, 1980; Little *et al.*, 1980; Magnus, 1981). In each of these 3 studies a rapidly increasing incidence of malignant melanoma in the area under study is reported, with a doubling in incidence rate over each 7–9 years. If the genotype of the Australian and Scottish populations are indeed similar, it would appear that environmental factors in Queensland increase the current incidence of malignant melanoma 5–6 fold.

It will be seen from Table II that the ratio of affected females to males shows an excess of females in all decades. These ratios are in broad agreement with the figures recently published for England and Wales for the earlier period 1968–71, and add circumstantial evidence to the suggestion that in relatively low-incidence areas a hormonally dependent or related aetiological factor results in a higher incidence in premenopausal females than in males of the same age range (Lee & Storer, 1980).

The ratio of 2 females to one male is similar to that reported in other countries with a relatively low incidence of melanoma, whereas in countries such as Australia with a high incidence the two sexes tend to be affected equally (Lee & Storer, 1980). Comparison with figures collected in Norway for 1977 shows an incidence in males and females which is equal at $10{\cdot}9/10^5$ in 1977 and an increase of $\sim 7\%$ p.a. is reported (Magnus, 1981).

This contrasts strongly with the striking female male ratio in Scotland of 2·17%. It will be of interest to see whether or not this sex difference becomes less marked if the Scottish figures rise to levels approaching those for Norway. Table III illustrates the high preponderance of lesions on the lower leg in females. Once again this is a common finding in other large published series.

The work of Clark, Breslow, Balch and their associates in the past decade has greatly extended the volume of useful information, often of considerable prognostic significance, which can be obtained from light-microscopic examination of appropriate blocks of paraffin-embedded tissue (Clark *et al.*, 1969; Breslow, 1977; Breslow & Macht, 1978; Balch *et al.*, 1978, 1979, 1980). Clark has suggested that there are several different histogenetic precursors or lateral growth phases of the pre-invasive epidermal component of cutaneous malignant melanoma. The 4 main histogenetic varieties of melanoma currently recognised are lentigo maligna, superficial spreading, nodular and acral lentiginous. Of these, the commonest in large reported series is the superficial spreading variety, and it will be seen that the Scottish figures (48%) also show this trend (Table IV). It is of interest that a higher proportion of lentigo maligna melanomas were diagnosed in Queensland in 1977 than in 1966 (Little *et al.*, 1980). The 1977 figures from this Australian state are 15·6% in males and 14·1% in females, whereas the SMG figure for 1979 is 13%. There is strong circumstantial evidence that cumulative total lifetime exposure to solar radiation plays a significant role in the aetiology of lentigo maligna melanoma (McGovern *et al.*, 1980) whereas the available evidence for superficial spreading and nodular melanoma suggests that intermittent sun exposure and burning may be a more important aetiological feature (Lancet, 1981). Nodular malignant melanoma is a lesion with no residual evidence of any *in situ* or lateral growth phase in the epidermis, and only a vertically invasive component. Our current understanding of the biological processes underlying malignant transformation would suggest that this lesion is not *ab initio* an aggressive vertically invasive lesion, but rather that the rapid rate of tumour expansion in this situation has obliterated all evidence of an *in situ* or pre-invasive phase. This group, which generally forms the majority of deeply invasive poor-prognosis tumours, currently comprises 25% of the Scottish series.

It will be seen from Table V that in 1979 63 patients (24%) had tumours $\geqslant 4$ mm thick. It is now well established that there is an inverse linear relationship between tumour thickness and survival and figures from other studies suggest that the 5-year survival rate for patients with lesions of this thickness is only 38%, even after adequate and extensive surgical excision (Pondes *et al.*, 1981). This therefore is a group in which studies of appropriate adjuvant therapy are urgently needed.

This would appear to be the first occasion on which a population-based group has had accurate histogenetic typing and measurement of tumour thickness carried out at the time of diagnosis. This is significant, as in retrospective studies lack of provision of suitable blocks may reduce the accuracy of these assessments.

If present trends continue the SMG will have accumulated extensive clinical and pathological information on 2500 patients with malignant melanoma by 1990. Much basic information on the incidence, morbidity and modes of treatment of this tumour will have been collected, and careful analysis of this data should allow valid conclusions to be reached about changes in its incidence, management, and behaviour in the Scottsh population.

All population figures in this paper are taken from the Annual Return of the Registrar General for Scotland, December 1979.

The authors wish to acknowledge with grateful thanks the collaboration of the many clinicians and pathologists in Scotland who have made material available to the SMG. This work is supported by a grant from the Chief Scientist for Scotland, to whom we also express our gratitude.

Members of the Central Committee of the Scottish Melanoma Group include R. M. MacKie, J. A. A. Hunter, K. C. Calman, A. C. H. Watson, J. MacGillivray, A. J. Carr, H. Crum, F. M. McGregor and I. Kemp.

### REFERENCES

BAKOS, L. & MACMILLAN, A. L. (1973) Malignant melanoma in East Anglia, England: An 11-year survey by site and type. *Br. J. Dermatol.*, **88**, 551.

BALCH, C. M., MURAD, T. M., SOONG, S. J., INGALLS, A. L., HALPERN, N. B. & MADDOX, W. A. (1978) Multifactorial analysis of melanoma: Prognostic histopathological features comparing Clark's and Breslow's staging methods. *Ann. Surg.*, **178**, 732.

BALCH, C. M., MURAD, T. M., SOONG, S., INGALLS, A. L., RICHARDS, P. C. & MADDOX W. A. (1979) Tumour thickness as a guide to surgical management of clinical Stage I melanoma patients. *Cancer*, **43**, 883.

BALCH, C. M., WILKERSON, J. A., MURAD, T. M., SOONG, S., INGALLS, A. L. & MADDOX, W. A. (1980) The prognostic significance of ulceration of cutaneous melanoma. *Cancer*, **45**, 3012.

BRESLOW, A. (1977) Problems in the measurement of tumour thickness and level of invasion in cutaneous melanoma. *Hum. Pathol.*, **8**, 1.

BRESLOW, A. & MACHT, S. D. (1978) Evaluation of prognosis in Stage I cutaneous melanoma. *Plast. Recons. Surg.*, **61**, 342.

CLARK, W. H., FROM, L., BERNARDINO, E. H. & MIHM, M. C. (1969) The histogenesis and biological behaviour of primary human malignant melanoma of the skin. *Cancer Res.*, **29**, 705.

ELWOOD, J. M., LEE, J. A. H., WALTER, S. D., MO, T., & GREEN, A. E. S. (1974) Relationship of melanoma and other skin cancer mortality to latitude and ultraviolet radiation in the United States and Canada. *Int. J. Epidemiol.*, **3**, 325.

HOLMAN, C. D. J., MULRONEY, C. D. & ARMSTRONG, B. K. (1980) Epidemiology of pre-invasive and invasive malignant melanoma in Western Australia *Int. J. Cancer*, **25**, 317.

HOUGHTON, A., FLANNERY, J. & VIOLA, M. V. (1980) Malignant melanoma in Connecticut and Denmark. *Int. J. Cancer*, **25**, 95.

LANCET (1981) Leading article: *The aetiology of melanoma. Lancet*, i, 253.

LANE BROWN, M. M., SHARPE, C. A. B., MCMILLAN, C. S. & MCGOVERN, V. J. (1971). Genetic predisposition to malignant melanoma and other skin cancers in Australia. *Med. J. Aust.*, **1**, 852.

LANE BROWN, M. M. & MELIA, D. F. (1973) Celticity and cutaneous malignant melanoma in Massachussetts. In *Pigment Cell: Mechanisms in Pigmentation*, Vol. 1 (Eds. McGovern and Russell). Basel: Karger. p. 229.

LEE, J. A. H. & CARTER, A. P. (1970) Secular trends in mortality for malignant melanoma. *J. Natl Cancer Inst.*, **45**, 91.

LEE, J. A. H. & STORER, B. E. (1980) Excess of malignant melanoma in women in the British Isles. *Lancet*, ii, 1337.

LITTLE, J. H., HOLT, J. & DAVIS, N. (1980) Changing epidemiology of malignant melanoma in Queensland. *Med. J. Aust.*, **1**, 66.

MCCARTHY, W. H., BLACK A. L. & MILTON G. W. (1980) Melanoma in New South Wales. *Cancer*, **46**, 427.

MCGOVERN, V. J., SHAW, H. M., MILTON, G. W. & FARGO, G. A. (1980) Is malignant melanoma arising in Hutchison's melanotic freckle a separate disease entity ? *Histopathology*, **4**, 235.

MAGNUS, K. (1981) Habits of sun exposure and risk of melanoma. *Cancer*, **49**, 2329.

PONDES, S., HUNTER, J. A. A., WHITE, H., MCINTYRE, M. A. & PRESCOTT, R. (1981) Cutaneous malignant melanoma in S. E. Scotland. *Q. J. Med.*, **50**, 103.

SCHREIBER, M. M., BOZZO, P. D. & MOON, T. E. (1981) Malignant melanoma in southern Arizona: Quadrupling incidence in decade 1969–78. *Arch. Dermatol.*, **117**, 6.

SVERDLOW, A. J. (1979) Incidence of malignant melanoma of the skin in England and Wales and its relationship to sunshine. *Br. Med. J.*, ii, 1324.

TEPPO, L., PAKKANEN, M. & HAKULINEN, T. (1978) Sunlight as a risk factor of malignant melanoma of the skin. *Cancer*, **41**, 2018.

WATERHOUSE, J. A. H., HUNT, C., TROUT, K. & 5 others (1976) *Cancer Incidence in Five Continents.* Vol. 3. Lyons: ARC. p. 453.